# Comparing Surgical Stress in Children Undergoing Open and Laparoscopic Inguinal Hernioplasty—A Single Center’s Prospective Study Results

**DOI:** 10.3390/children12121588

**Published:** 2025-11-22

**Authors:** Charikleia Demiri, Ioannis Spyridakis, Stavros Iliadis, Anastasia Giannakou, Maria Bantadaki, Christos Kaselas

**Affiliations:** 1 Second Department of Paediatric Surgery, Papageorgiou General Hospital of Thessaloniki, “Aristotle” University Medical School, Ring Road of Thessaloniki, St Paul Area, 56429 Thessaloniki, Greece; ispyrida@auth.gr (I.S.); xkaselas@auth.gr (C.K.); 2 Department of Biological Chemistry, Medical School, Faculty of Health Sciences, Aristotle University of Thessaloniki, 54124 Thessaloniki, Greece; siliad@auth.gr; 3 Department of Immunology and Histocompatibility, Papageorgiou General Hospital of Thessaloniki, Ring Road of Thessaloniki, St Paul Area, 56429 Thessaloniki, Greece; giannakouan@gmail.com (A.G.); mariampantadaki@gmail.com (M.B.)

**Keywords:** pediatric surgery, inguinal hernia, laparoscopic unilateral inguinal hernioplasty, open unilateral inguinal hernioplasty, surgical stress, biomarkers, follow-up

## Abstract

**Highlights:**

- Surgical stress is probably an underestimated factor in minimal invasive pediatric surgery, contrary to adults.
- Laparoscopic hernioplasty results in earlier and abrupt release of stress biomarkers.

**What are the main findings?**

- Levels of albumin, C-reactive protein, malondialdehyde (MDA), cortisol, and ferritin did not differ significantly between the open and laparoscopy techniques.
- Time-course profile of WBC levels over time differed between the surgery groups, with the laparoscopic group having slightly higher post-operative WBC levels and the open surgery group having a similar but delayed temporal profile.

**What are the implications of the main findings?**

- Both surgical techniques have a similar impact on the inflammatory response in children, indicating that laparoscopy is a safe and effective alternative to open hernioplasty regarding operative stress.
- When counseling children’s families with inguinal hernias, either approach is acceptable; laparoscopy is safe in terms of early stress biomarkers.

**Abstract:**

Background: Inguinal hernioplasty in the pediatric population is a common minor injury operation. Whether the surgical approach alters perioperative stress responses remains uncertain. The aim of this study was to compare early inflammatory and endocrine stress markers after open (OIH) and laparoscopic inguinal hernioplasty (LIH). Methods: A single-center prospective observational study with 1:1 allocation between 2021 and 2023. Otherwise healthy children, scheduled for elective unilateral indirect inguinal hernia repair, were assigned to open or laparoscopic hernioplasty. Blood samples were collected at five time points, measuring levels of white blood cells, cortisol, MDA, ferritin, albumin, and CRP. Analyses used two-way repeated measures ANOVA with Greenhouse–Geisser correction and Bonferroni-adjusted planned contrasts at 24 h. Results: Thirty-two children aged from 2.4 months to 11 years with a mean age of 3.8 years (± 2.46 Standard Deviation, SD) were randomized equally. Operative times were longer in the laparoscopic group [01:07 (±00:20-SD)]; [open group operative duration: 00:41 (±00:16-SD), (*p* < 0.01)]; discharge on postoperative day 1 was universal. The group × time interaction was not significant for CRP, cortisol, MDA, albumin, or ferritin. WBC showed a modest interaction consistent with an earlier postoperative rise after laparoscopy (*p* = 0.006). No surgical site infections or recurrences occurred over a 2-year follow-up. Conclusions: This study demonstrates that LIH and OIH in children have a comparable impact on the inflammatory response. LIH is a safe and effective alternative to the traditional open repair method regarding operative stress. To validate these findings and assess the long-term implications of each surgical approach on children’s pathophysiology, further research is warranted.

## 1. Introduction

Surgical stress denotes the body’s response to surgical intervention, initiating a cascade of pathophysiological and immunological reactions aimed at maintaining homeostasis and promoting survival [1,2]. Stress response, triggered by tissue trauma, varies based on the operation type, patient’s comorbidities, genetic predisposition, tissue handling, and anesthetic management [1].

Pediatric patients appear to be notably vulnerable to surgical stress response owing to reduced energy reservoir, high brain-to-body ratio, and dependence on glucose [2,3,4,5,6,7,8]. Operative stress in children has received limited investigation to date, in contrast to numerous studies comparing open and laparoscopic surgery–induced stress responses in adults [8,9,10,11,12,13,14,15].

Inguinal hernia repair, though a minor injury operation, is common in pediatric surgery and ideal for studying surgical stress [15]. Current studies mainly compare surgical outcomes rather than pathophysiological impacts [15]. Both open and laparoscopic techniques are similar in terms of intraoperative injury, bleeding, surgery duration, hospitalization, hernia recurrence, postoperative testicular atrophy, and hydrocele occurrence [16]. The laparoscopic approach allows better control of the contralateral inguinal orifice by reducing the risk of metachronous hernias or hydroceles [16].

Regular blood markers for assessing surgical stress include cortisol, interleukin (IL)-6, C-reactive protein (CRP), and white blood cell (WBC) count [17]. Less common markers in children include albumin, malondialdehyde (MDA), and ferritin [15]. These biomarkers rise at different postoperative time points, reflecting the severity and nature of surgical stress [2,3,17,18,19,20,21,22,23].

We conducted a prospective observational study to test whether the surgical approach modifies early inflammatory and endocrine stress response during pediatric inguinal hernia repair.

## 2. Materials and Methods

### 2.1. Study Design

Prospective, parallel-group observational study at a single tertiary pediatric surgical unit, approved by the Local Ethical Committee (#4.295/4/26.01.2021); informed written consent was obtained from each patient’s parent or legal guardian. Eligible participants were children undergoing elective unilateral indirect inguinal hernioplasty. Patients with comorbidities affecting immunity, recurrent or bilateral hernia, emergency surgery, and chronic medication use were excluded.

Children were admitted 24 h preoperatively. Medical history and demographic data (age, sex, body mass index, and hernia lateralization) were recorded. Participants fasted for six hours preoperatively, receiving a maintenance infusion of dextrose/saline. Pre-medication was administered to alleviate anxiety. Operating room temperature was maintained between 27 and 29 °C. All procedures were conducted under general anesthesia with endotracheal intubation, following a standard induction protocol, performed or supervised by two consultant pediatric surgeons. Each child received two standard doses of antibiotics. Postoperative pain relief was managed with standard intravenous administration of paracetamol (15 mg/kg per dose).

The duration from anesthesia induction to reversal and from first to last cut was recorded. Blood samples were taken 24 h preoperatively (time point 1), immediately after anesthesia induction (time point 2), immediately after the last cut (time point 3), and at 6 (time point 4) and 24 h postoperatively (time point 5) to measure levels of white blood cells (WBC), albumin (ALB), C-reactive protein (CRP), malondialdehyde (MDA), cortisol (COR), and ferritin (FER). The length of stay was one day, while the follow-up duration was two years.

### 2.2. Sample Size Calculation

The sample size for this study was determined using data from Jucik et al.’s research, titled “Comparison of Inflammatory Stress Response Between Laparoscopic and Open Approaches for Pediatric Inguinal Hernia Repair” [14]. This study is recognized in the literature as the only published work addressing a comparable research question.

They reported a mean WBC of 11.4 ± 3.1 vs. 7.6 ± 1.6 × 10^9^/L. The implied effect size (Cohen’s d) is ≈1.54 (pooled SD ≈ 2.47). For a two-sided α = 0.05 and 80% power, a two-sample comparison requires ≈14 participants in total (equal allocation), as computed with OpenEpi. We prospectively targeted a larger cohort and enrolled 32 children, exceeding the minimum to preserve ≥80% power and to support repeated-measures analyses.

### 2.3. Measurement of the Inflammatory Markers

Blood samples were collected by a pediatric surgery resident experienced in venipuncture. Immediately upon collection, the samples were analyzed at the laboratory of the tertiary hospital where the study took place. Plasma serum, after centrifugation at 3000 rpm, was stored at −72 °C for subsequent MDA analysis in the Laboratory of Biochemistry of “Aristotle” University Medical School.

WBC (K/μL) and their subpopulations were assessed using laser flow cytometry with optical scattering technology and hydrodynamic focusing. Plasma levels of ALB (g/dL), FER (ng/mL), and COR (μg/dL) were measured using chemiluminescence on an automated analyzer. CRP levels (mg/dL) were quantified using nephelometry on an automated analyzer. MDA levels (ng/mL) in plasma serum were determined using differential spectrophotometry, by analyzing the thiobarbituric acid–malondialdehyde complex within the wavelength range of 400–650 nm.

### 2.4. Operative Technique Description

#### 2.4.1. Open Technique

The patient is placed in the supine position with both arms secured at their sides. An inguinal incision is made horizontally through the aponeurosis of the external oblique abdominal muscle, 1 cm above the external inguinal ring. This incision, approximately 3 cm in length, extends along the inguinal canal to the internal inguinal ring. The cord structures are carefully separated from the hernia sac while preserving the transverse fascia intact. In female patients, the sac is carefully dissected from the surrounding structures of the canal of Nuck. The sac is then opened to confirm the absence of ovarian content before proceeding with ligation. The hernia sac is securely ligated using two absorbable sutures of Vicryl 3-0 (Ethicon ^®^). A portion of the peripheral hernia sac is subsequently excised. Finally, the tissues are reconstructed in their anatomical layers using Vicryl (Ethicon^®^) sutures, while the skin is approximated with subcuticular stitches.

#### 2.4.2. Laparoscopic Technique

The patient is placed in the supine position with both arms secured at their sides. Using a Hasson technique for the umbilical approach, a 5 mm trocar is inserted to create pneumoperitoneum with pressures and flows appropriate for the patient’s age. Two 3 mm working trocars are placed in the right and left lateral abdomen at the level of the umbilicus. Then, the patient is positioned in the Trendelenburg position. The internal inguinal ring is identified, and the parietal peritoneum is grasped and incised approximately 0.5 cm from the internal inguinal ring. In female patients, the above step is replaced by grasping, coagulating, and cutting the round ligament of the uterus.

Next, the neck of the hernia sac is circumferentially dissected around the internal inguinal ring. Once the neck of the hernia sac is fully dissected, it is grasped and carefully separated from the surrounding inguinal canal structures. A portion of the sac is then transected and removed through the 3 mm trocar.

The posterior wall of the inguinal canal is narrowed with stitches [Ticron 2-0 sutures (Covidien ^®^)] placed between the conjoint tendon and the iliopubic tract. Finally, the parietal peritoneum is closed circumferentially around the internal inguinal ring. The trocars are removed, and the pneumoperitoneum is deflated. The umbilicus is closed with Vicryl (Ethicon^®^) sutures, while the skin is approximated with subcuticular stitches.

### 2.5. Statistical Analysis

Statistical processing of demographic data was carried out using SPSS v.26 (Statistical Package for the Social Sciences) v.26 (SPSS, Inc., Chicago, IL, USA). As all the demographic parameters predominantly follow the normal distribution and based on the central limit theorem, the parametric tests, independent sample t-test, and one-way and two-way repeated measures ANOVA, with significance at 0.05, were used for statistical analysis of the results. The two-way repeated measures ANOVA was used to examine the effects of time and surgical method on inflammation biomarkers. The statistical tests included Wilks’ Lambda and Mauchly’s Test of Sphericity to account for violations of sphericity; the Greenhouse–Geisser metric was preferred when sphericity was violated.

## 3. Results

### 3.1. Sample Demographics

**Figure 1:**
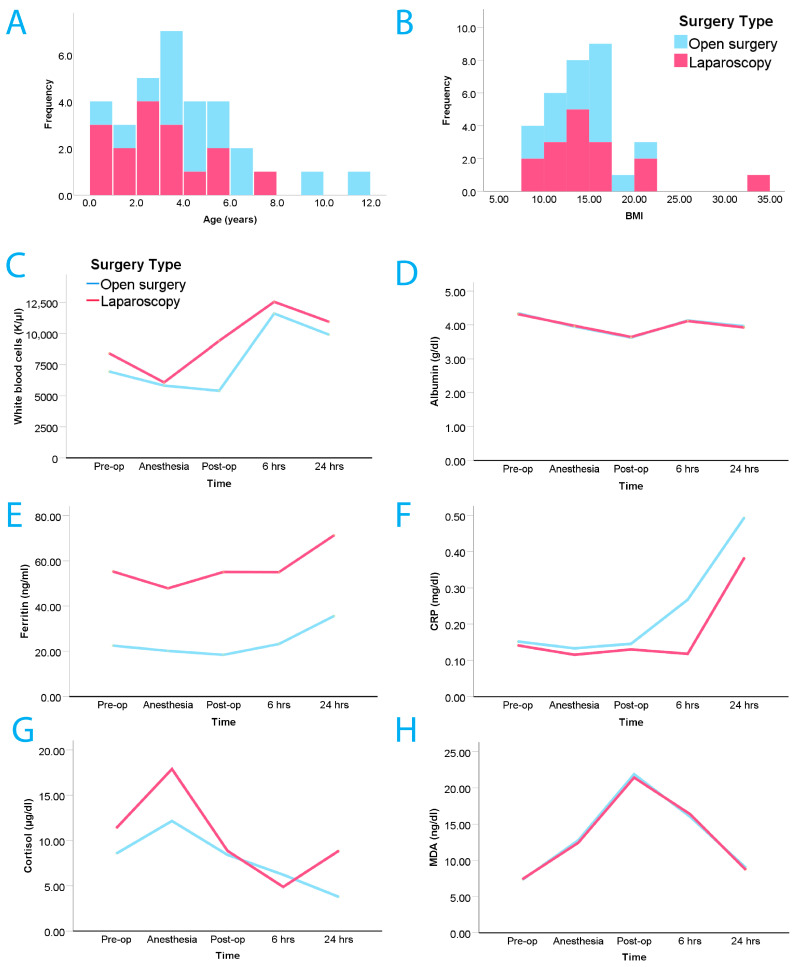
(**A**) Age distribution per surgery type group; (**B**) BMI distribution per surgery type group. (**C**) white blood cell count (WBC); (**D**) albumin; (**E**) ferritin; (**F**) C-reactive protein (CRP); (**G**) cortisol; (**H**) malondialdehyde (MDA). Inflammatory markers at the five different time points for both surgery groups. Produced using Adobe Illustrator CC 2017 version.

The final group included 32 children scheduled for elective inguinal hernioplasty. They were randomized into two main study groups (open and laparoscopic surgical treatment groups). Of the 32 children, 24 (75%) were boys. In 21 patients (65%), the inguinal hernia was on the right side. The two surgical repair groups had the same size of 16 patients. Age ranged from 2.4 months to 11 years, with a mean age of 3.8 years (±2.46 Standard Deviation, SD). A notable age difference was only present in the LIH group (the boys’ mean age was 2.2 years (±1.10 SD), while the girls were older at 4.4 years (±2.40 SD), with *p* < 0.05). The mean body mass index (BMI) was 7.68 (±4.94) (Figure 1). Detailed descriptive demographics of the patients by surgical group are shown in Table 1. Table 2 depicts all inflammatory markers for all stages per surgery group.

### 3.2. Operative Duration

The mean total operational duration (from anesthesia induction to reversal) was 01:12 (h:mins) in the open group and 01:58 in the laparoscopy group, while the mean operative duration (from first to last cut) was 00:41 and 01:07, respectively. The above duration difference between the open and laparoscopy group was statistically significant (*p* < 0.001), with the LIH group presenting a longer operative time duration.

### 3.3. Inflammatory Biomarkers

Inflammatory markers were measured at the above-described five different time points. There was a single statistical difference, within the LIH group, between girls and boys in all biomarkers, and that was at the pre-operative cortisol levels [boys’ mean level: 13.28 μg/dL (±4.88 SD) vs. girls’ mean level: 7.09 μg/dL (±3.63 SD) with *p* < 0.05]. Figure 1 illustrates all the marker levels across the time points for both groups.

For WBC levels, a significant temporal main effect was observed across all time points (Wilks’ Lambda (λ) = 0.137, F (4, 27) = 42.367, *p* < 0.001), indicating a non-linear active response to inflammation for WBC levels over time. WBC levels fluctuate according to the operative phase and are in line with the expected inflammation levels. There was also a statistically significant time by surgery interaction effect (λ = 0.597, F (4, 27) = 4.565, *p* = 0.006). This interaction indicates that the time-course profile of WBC levels over time differed between the surgery groups, with the laparoscopic group generally having slightly higher post-operative WBC levels and the open surgery group presenting a similar but delayed temporal profile, illustrated in Figure 1 by the phase shift evident in the relevant line plot.

ALB levels also had significant temporal variability (λ = 0.073, F (4, 27) = 85.987, *p* < 0.001) across the five measurement points. However, there was no significant interaction between time and surgical method (λ = 0.951, F (4, 27) = 0.347, *p* = 0.844), indicating that the type of surgery did not significantly alter the time-course profile of ALB levels.

FER levels showed significant time effects (λ = 0.0.369, F (4, 27) = 11.541, *p* < 0.001), but the interaction between time and surgery type was not significant (λ = 0.866, F (4, 27) = 1.045, *p* = 0.403).

For CRP, significant effects were observed over time (λ = 0.636, F (4, 27) = 3.871, *p* = 0.013), indicating temporal changes in CRP levels across the five measurement points. However, the interaction effect between time and surgery type was not significant (λ = 0.877, F (4, 27) = 0.950, *p* = 0.451), suggesting that the observed temporal changes did not differ significantly between the open surgery and laparoscopic groups.

COR levels also displayed significant temporal changes (λ = 0.448, F (4, 27) = 8.333, *p* < 0.001), with no significant interaction between time and surgery type (λ = 0.808, F (4, 27) = 1.605, *p* = 0.202). The analysis of MDA levels indicated significant main effects over time (λ = 0.020, F (4, 27) = 333.515, *p* < 0.001), with no significant interaction effect between time and surgery (λ = 0.854, F (4, 27) = 1.151, *p* = 0.354).

While the biomarkers CRP, MDA, COR, and FER showed significant changes over time, the type of surgery did not significantly alter the overall patterns of these changes. However, surgical technique did appear to influence the temporal profile of WBC levels, with laparoscopic techniques eliciting a sharper response and open techniques evoking a similar, albeit delayed-phase response. Table 3 provides a summary of all key statistical metrics for all inflammation markers.

Levels of hemoglobin were compared between time points 2 and 5. It was found that they did not differ statistically (paired *t*-test, *p* = 0.488) for all children, proving that the technique and quantity of blood used for sampling collection were safe for the participants.

## 4. Discussion

Surgical stress has been extensively studied, given its direct relevance to the management of patient pathophysiology [19]. To our knowledge, this is the first study that compares surgical stress between open and laparoscopic hernioplasty in children of both sexes.

In children, surgical procedures are influenced by multiple stressors, including emotional factors, pain control, tissue injury, bacterial contamination, drug administration, fasting, and temperature fluctuations [2,24]. In response to these stressors, the body activates a series of protective and adaptive mechanisms that serve to support homeostasis [2].

The endocrine component of stress response is characterized by the release of hormones, including catecholamines, cortisol, and glucagon, which mobilize proteins, fats, and carbohydrates to support energy production during the perioperative period [25]. WBC level release is an integrated part of the inflammatory response, with granulocytosis and lymphopenia reaching the maximum level within 6 h after a skin incision [26].

Cortisol levels rise the first 4 to 6 h after the first cut, while CRP reaches its peak value during the first and second postoperative day, proportionally to the surgical complexity [17,18]. Regarding albumin, Hübner et al. concluded that its postoperative drop can evaluate the surgical stress magnitude in adults [27]. Moreover, Gabay et al. indicated that albumin levels decrease, while CRP and FER rise in response to the immune reaction [20]. As phenomena that develop during the inflammatory response, the same article describes, among others, leukocytosis and an increase in plasma cortisol [20].

In our study, we found that cortisol levels tend to increase during the operation, both in the laparoscopic and open groups, but decrease immediately after it. There was no statistically significant difference between the OIH and LIH regarding the fluctuation of cortisol levels. Our finding is in line with the literature since, in children, particularly in newborns, the endocrine response to surgical stress is characterized by a rapid increase in stress hormones with greater variability [28]. Typically, plasma levels of catecholamines and cortisol are elevated during surgery but tend to return to baseline levels in the early postoperative period [18]. A similar study in adult hernioplasty also found a non-statistically significant difference in cortisol values between groups, concluding that the two approaches do not affect, in a different way, the cortisol levels [29]. Although our study found no significant difference in cortisol levels between the groups, it is important to assess cortisol alongside other inflammatory markers when comparing the surgical stress of these procedures, since cortisol levels can be influenced by various factors, including emotional stress, beyond surgical trauma. The lack of difference in our participants’ cortisol levels might be attributed to factors such as preoperative stress alleviation through premedication, the relatively minor nature of hernioplasty in children, and the systematic administration of painkillers postoperatively.

Our LIH group exhibited slightly higher post-operative WBC levels, while the OIH group showed a similar pattern, albeit with a delayed onset. In the study of Jukic et al., there is a significant difference in the mean values of WBC between the open and laparoscopic groups of inguinal hernia repair, while the same was found in the study of Redmond et al., when they studied the immune function in adult patients undergoing open versus laparoscopic cholecystectomy [14,30]. Our findings differ from the existing literature, as we observed that WBC counts increased more rapidly and to a higher degree in the laparoscopy group compared to the open group. This discrepancy might be explained by the more complex laparoscopic technique employed in our study, which involved a longer duration of pneumoperitoneum exposure compared to the technique used in Jukic et al.’s trial. Additionally, this difference could be due to the more frequent blood sampling in our study, particularly around the time of surgery, as opposed to the time points used in Jukic et al.’s study, where WBC counts were measured one day preoperatively, 24 h, and seven days postoperatively. As a result, significant fluctuations in WBC counts around the time of surgery may not have been captured in their study.

Regarding albumin levels, the pattern of its measurements in our study was exactly the same, having no difference both during the different time points and between the two surgical groups. This can be explained by the short period of fasting preoperatively, oral feeding initiation three hours postoperatively, and that children underwent a minor injury operation of hernioplasty, which prevents the exacerbated catabolism of proteins for acute phase proteins production.

Mean CRP levels of both groups did not differ preoperatively. It should be noted that the mean CRP level was higher 6 h postoperatively in the open group. While the mean CRP level was higher 24 h postoperatively in the laparoscopy group, there was no statistically significant difference between the mean CRP levels between the two groups. A study by Li et al. aimed to evaluate laparoscopic appendectomy (LA) in comparison with conventional open appendectomy (OA) in children [9]. Preoperative CRP levels were not different between the two groups, but the rise (preoperative vs. postoperative) of CRP in the LA group was remarkably less steep than in the OA group 12 h postoperatively [9]. Sekhon et al., in their study, comparing surgical stress among open and laparoscopic nephrectomy in children, noticed that the acute rise in CRP 24 h postoperatively in the open group was significantly higher when compared to both the preoperative and 4 hours’ postoperative values, while this rise was not statistically significant when compared to the 24 hours’ postoperative value in the laparoscopy group. Furthermore, in the study by Jukic et al., when the inflammatory response was compared between open and laparoscopic hernioplasty in children, CRP levels showed a more pronounced elevation in the cohort undergoing the open procedure 24 h postoperatively [14]. The discrepancy between our findings of higher, though not statistically significant, mean CRP values at 24 h postoperatively in the laparoscopy group, and the results reported in the literature may be attributed to differences in the laparoscopic techniques used. Our approach may be slightly more invasive compared to those employed by Jukic et al. [14]. Additionally, Li et al. observed lower mean CRP levels in their laparoscopy group at 12 h postoperatively, a finding consistent with our results at 6 h postoperatively [9]. Regarding the comparison between our study and that of Sekhon et al., which suggests the superiority of laparoscopic nephrectomy in children, it is important to note that nephrectomy is a more complex procedure than hernioplasty [11]. Therefore, the lower mean CRP levels reported in the laparoscopic nephrectomy group in Sekhon et al.’s study may not be directly comparable to our findings.

MDA is employed to assess oxidative stress, a key component of the overall surgical stress response [21]. The use of carbon dioxide (CO_2_) during laparoscopy may lead to an increase in CO_2_ concentration in tissues, potentially altering the extent of free radical-induced injury. This interaction occurs because CO_2_ can react with peroxynitrite, a reactive nitrogen species, which may influence the overall oxidative stress and tissue damage during the procedure [31]. The mean MDA values in both our groups exhibited a similar pattern of increase and decrease, showing no significant differences either preoperatively, postoperatively, or at various time points during the study. Since MDA serves as an indicator of oxidative stress, which is part of the overall surgical stress response, these findings suggest that oxidative stress levels are comparable between open and laparoscopic hernioplasty procedures in children. This observation aligns with the results of McHoney et al., who found no significant difference in inflammatory and immune responses between open and laparoscopic Nissen fundoplication in infants and children [10]. However, trials in adult populations undergoing open and laparoscopic inguinal hernia correction demonstrated a statistically significant elevation in MDA levels in the open treatment groups [32]. Our findings on MDA could be explained firstly by the low-pressure pneumoperitoneum used in children, contrary to adults, according to their age, and secondly by the absence of comorbidities in our groups, facts that may provoke a significantly lower free radical-induced injury.

Ferritin, widely utilized in clinical practice to assess body iron stores, is also classified among acute-phase proteins, which exhibit elevated plasma concentrations during inflammatory responses [20]. Plasma ferritin serves as a key biomarker of inflammation, being released from the intracellular compartment into the circulation following cellular injury [22]. The cellular damage incurred during surgical procedures underlies the use of changes in plasma ferritin levels as an indicator for evaluating surgical stress [23]. In our research, mean ferritin values were statistically significant across the five time points, but there were no significant differences between the two groups. Notably, preoperative mean ferritin levels were higher in the laparoscopy group, which may be attributed to the inclusion of two infants in this group who had ferritin levels exceeding 150 ng/mL, a normal range for their age group. To our knowledge, no other comparative studies in children have assessed ferritin levels in this context. However, a 2022 study in the literature used ferritin as an inflammatory marker to compare open and laparoscopic cholecystectomy in adults. This study found higher ferritin levels in the group undergoing open surgery [33].

Our study has limitations. Firstly, this is a small study conducted at a single center with a limited number of 32 patients. Secondly, our trial studies a minor injury operation, as open and laparoscopic inguinal hernioplasty is considered in the literature. Thirdly, the open and laparoscopic hernioplasty techniques employed in our study differed regarding the posterior inguinal canal wall reinforcement step, which was followed only in the laparoscopy group. Fourthly, our study comprised both girls and boys, but the girls were only 25% of the total participants. Last but not least, the participants had no comorbidities, a fact that could influence the results.

## 5. Conclusions

In our study, slight differences in the mean levels of inflammatory markers were observed across the five different time points of measurement. However, there was no statistically significant difference in the mean values of these markers between the open and laparoscopic hernioplasty groups apart from WBC levels. Our findings suggest that both surgical techniques have a similar impact on the inflammatory response in children, indicating that laparoscopy is a safe and effective alternative to open hernioplasty. The initial assumption that was aimed to be tested, that laparoscopic inguinal hernia repair in children results in less operative stress release than the open repair, is not confirmed according to our findings. When counseling families of little patients, either approach is acceptable; laparoscopy is safe but not necessary or superior in terms of early stress biomarkers. Nonetheless, further research is needed to strengthen the validity of these results.

## Figures and Tables

**Table 1:** Patient demographic and clinical data.

		Treatment Group				
		OIH		LIH		
		N	N%	N		N%
Sex	Male	14	87.5%	10		62.5%
	Female	2	12.5%	6		37.5%
Surgery Side	Left	6	37.5%	5		31.3%
	Right	10	62.5%	11		68.8%
		OIH		LIH		Independent sample -*t* test
		Mean	SD	Mean	SD	*p* Value
Age (years)		4.63	2.70	3.03	1.98	0.065
Weight (kg)		21.49	9.50	14.31	5.81	0.015
Height (m)		1.20	0.20	1.00	0.29	0.027
BMI(kg/m^2^)		14.55	3.48	15.35	6.17	0.653
Hb after anesth induction		11.82	1.18	11.45	1.23	0.488 ^#^
Hb 24 h Post-op		11.68	0.92	11.35	0.99	
WBC Pre-op (K/μL)		6955.00	1936.57	8395.00	2058.62	0.05
Albumin Pre-op (g/dL)		4.34	0.22	4.32	0.28	0.800
Ferritin Pre-op (ng/mL)		22.47	9.93	53.37	84.14	0.165
CRP Pre-op (mg/dL)		0.15	0.05	0.15	0.06	1
Cortisol Pre-op (μg/dL)		8.57	2.75	10.96	5.32	0.120
MDA Pre-op (ng/dL)		7.31	1.32	7.56	1.44	0.614
Total Operational Duration (h: mins)		01:12	00:17	01:58	00:19	<0.001
Operative duration (h: mins)		00:41	00:16	01:07	00:20	<0.001

^#^ paired *t*-test.

**Table 2:** Inflammatory markers at the five different time points for both surgery type groups. Time point 1: 24 h preoperatively; time point 2: immediately after anesthesia induction; time point 3: immediately after the last cut; time point 4 and 5: 6 and 24 h postoperatively, respectively.

Biomarker	Units of Measurement	Biomarker Normal Range	Time Point	OIH Mean (SD)	LIH Mean (SD)	*p*-Value * (Between Time Points)	*p*-Value * (Between Groups)
WBC	K/μL	4000–11,000	1	6.955 (1.937)	8.395 (2.059)	<0.001	0.006
			2	5.819 (1.860)	6.143 (2.030)		
			3	5.402 (1.894)	9.422 (4.110)		
			4	11.603 (3.419)	12.691 (4.246)		
			5	9.890 (2.398)	11.007 (3.556)		
Albumin	g/dL	3.80–5.40	1	4.34 (0.22)	4.32 (0.28)	<0.001	0.844
			2	3.95 (0.24)	3.98 (0.28)		
			3	3.62 (0.26)	3.65 (0.31)		
			4	4.13 (0.22)	4.10 (0.31)		
			5	3.96 (0.24)	3.90 (0.35)		
Ferritin	ng/mL	10–405	1	22.47 (9.93)	53.37 (84.14)	<0.001	0.403
			2	20.12 (9.22)	45.89 (66.60)		
			3	18.36 (9.71)	52.58 (71.55)		
			4	23.18 (10.49)	52.89 (67.29)		
			5	35.66 (14.75)	69.63 (70.44)		
CRP	mg/dL	<0.5	1	0.15 (0.05)	0.15 (0.06)	0.013	0.451
			2	0.13 (0.04)	0.12 (0.04)		
			3	0.15 (0.06)	0.13 (0.05)		
			4	0.27 (0.28)	0.13 (0.08)		
			5	0.49 (0.27)	0.83 (1.82)		
Cortisol	μg/dL	5.27–22.45	1	8.57 (2.75)	10.96 (5.32)	<0.001	0.202
			2	12.15 (6.47)	17.36 (7.82)		
			3	8.41 (5.58)	8.55 (7.22)		
			4	6.22 (7.14)	4.66 (9.05)		
			5	3.77 (4.17)	8.55 (9.43)		
MDA	ng/dL	<0.001076	1	7.31 (1.32)	7.56 (1.44)	<0.001	0.354
			2	12.77 (0.88)	12.63 (2.05)		
			3	21.88 (1.85)	21.61 (2.65)		
			4	16.03 (1.70)	16.44 (2.06)		
			5	8.98 (1.10)	8.60 (1.29)		

* GLM two-way repeated measures ANOVA.

**Table 3:** Κey statistical parameters from the two-way repeated measures ANOVA for each biomarker, including Wilks’ Lambda (λ), the F-statistic with degrees of freedom (df1, df2), *p*-value, and Partial η^2^ (eta squared), which indicates the effect size.

Biomarker	Wilks’ λ	F (df1, df2)	*p*-Value	Partial η^2^
WBC	0.137	42.367 (4, 27)	<0.001	0.863
Albumin	0.073	85.987 (4, 27)	<0.001	0.927
Ferritin	0.195	27.624 (4, 27)	<0.001	0.805
CRP	0.636	3.871 (4, 27)	0.013	0.364
Cortisol	0.034	193.824 (4, 27)	<0.001	0.966
MDA	0.02	333.515 (4, 27)	<0.001	0.98

## Data Availability

The data presented in this study are available on request from the corresponding author.

## Abbreviations

The following abbreviations are used in this manuscript:

OIH	Open Inguinal Hernioplasty
LIH	Laparoscopic Inguinal Hernioplasty
WBC	White Blood Cells
CRP	C-Reactive Protein
MDA	Malondialdehyde
ALB	Albumin
COR	Cortisol
FER	Ferritin
IL-6	Interleukin-6
